# Tungsten-based Ultrathin Absorber for Visible Regime

**DOI:** 10.1038/s41598-018-20748-9

**Published:** 2018-02-05

**Authors:** Ahsan Sarwar Rana, Muhammad Qasim Mehmood, Heongyeong Jeong, Inki Kim, Junsuk Rho

**Affiliations:** 1 Department of Electrical Engineering, Information Technology University of the Punjab, Lahore, 54000 Pakistan; 2 Department of Mechanical Engineering, Pohang University of Science and Technology (POSTECH), Pohang, 37673 Republic of Korea; 3 Department of Chemical Engineering, Pohang University of Science and Technology (POSTECH), Pohang, 37673 Republic of Korea

## Abstract

Utilizing solar energy requires perfect absorption of light by the photovoltaic cells, particularly solar thermophotovoltaics (STPVs), which can be eventually converted into useful electrical energy. Ultrathin nanostructures, named metasurfaces, provide an intriguing platform to develop the miniaturized solar energy absorbers that can find potential applications in integrated photonics, optical sensing, color imaging, thermal imaging and electromagnetic shielding. Therefore, the quest of novel materials and designs to develop highly efficient absorbers at minuscule scale is an open topic. In this paper, novel absorbers using tungsten-metasurface are developed which give ultrahigh absorbance over a wide frequency spectrum. The proposed designs are two-dimensional, polarization insensitive, broadband and are predicted to give better response under high temperatures ascribed to high melting point of tungsten i.e. 3422 °C. Amongst these designs, cross alignment is found optimum for tungsten, because it is impedance matched with the free space for visible spectrum. This cross arrangement is further tweaked by changing width, height and length resulting in 7 different optimized solutions giving an average absorbance greater than 98%. One, amongst these solutions, gave a maximum average absorbance of 99.3%.

## Introduction

Modern society relies on power to function, which comes from different sources of energy. They can be grouped into two categories as ‘renewable’ and ‘non-renewable’. In the former category sun is one of the major sources of energy, the solar energy.

The solar energy can be captured by various mechanisms but one with the most potential is photovoltaic (PV) cells. Basic function of a PV cell is the generation of charge carriers which are then collected in external circuit, generating electric current^1^. On the other hand, solar energy can also be captured by the help of solar thermal photovoltaics (STPVs), which utilizes heat flow between hot and cold layer to generate electricity. For a STPV system to have high efficiency, there is a need of high absorbance of solar spectra i.e. absorbance of photons varying in energies to create an appreciable temperature difference between hot and cold layer^2–4^. This calls for a highly efficient solar absorber. Other applications of absorbers are found in optical sensing^5^, color imaging^6^, thermal imaging^6,7^, electromagnetic shielding^7^, etc.

The advent of sophisticated deposition techniques allowed fabrication of subwavelength structures i.e. Metamaterials (having lower dimensions than operating wavelength). These metamaterials (MMs) show variable properties than regular materials as they give freedom to alter permittivity of a material, which in turn gives varying refractive index thus achieving mutable response from the device. Manipulation of these metamaterials and metasurfaces (two-dimensional version of metamaterials) allow us to make different devices employing numerous phenomena which were impossible to observe using bulk materials like, optical couplers^8^, optical vortices^9,10^, orbital angular momentum (OAM) generation^11^, holograms^12–14^, optical cloaking^15^, tractor beam^16^ and PLAs (Perfect Light Absorbers).

The first mention of a “perfect absorber” came in 2008^17^, which showed a PLA in microwave regime at 11.5 GHz. Same group proposed a perfect absorber in visible region^18^. Since then, many broadband absorbers are sought with different structures employing the phenomenon of resonance or impedance matching^19^ to maximize absorbance over a specific wavelength.

Broadband PLAs are also established with a topology of metal-dielectric-metal layers. Broadband Structures previously demonstrated had different variations of simpler structures such as nanopillars made up of different variations of cylinders^20^, disks (cylinders)^21,22^, arrangement of crosses and cylinders^23^, nanopyramids (cones)^24–26^, rods (in shape of square)^27^, spheres, tetrahedral structure^28^, and even random deposition of particles^29^. A simple structure of square and in the end cross is used as a design in this research.

Major motivation behind the research is introduction of tungsten (*W*) for nanostructure layer which has higher melting point than any other metal i.e. Gold (Au), Silver (Ag), Chromium (Cr), Copper (Cu) and even Titanium Nitride (TiN) which is a refractory material. This high melting point of tungsten will help tungsten absorber to withstand high temperatures when absorbing photons of greater energy. Previous structures involving tungsten were made up in the shape of cones^25,26^, but the designs presented in this paper are 2D, as they are not varying in z-direction (height). The design presented in^22^ is also 2D but it is not designed for visible regime and it gives less absorbance. Since tungsten does not support surface plasmons in optical range, high absorbance is ascribed to impedance matching of tungsten absorber with the free space.

## Tungsten Based Absorber

**Figure 1:**
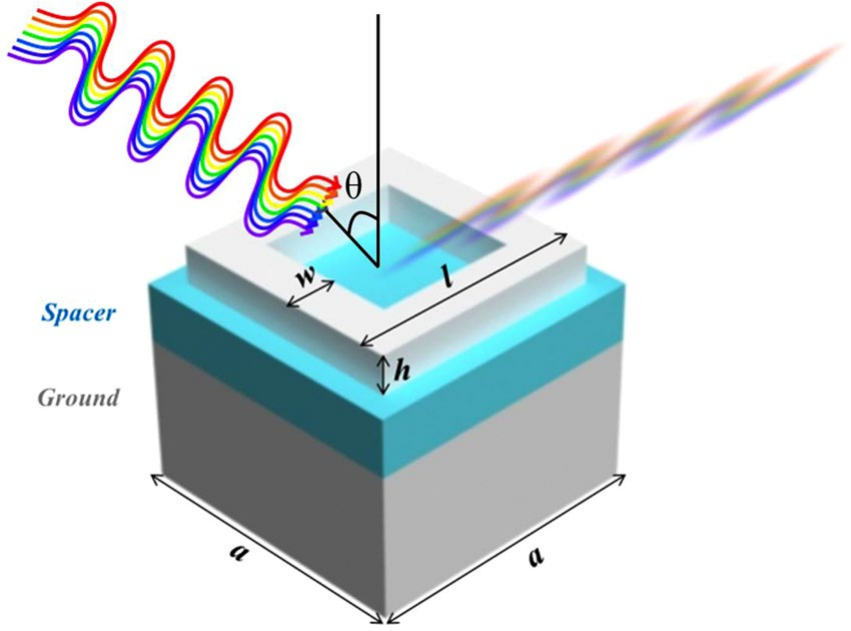
Square ring structure with *w* (width) = 50 nm, *h* (height) = 40 nm, *l* (length) = 250 nm, *a* (period) = 300 nm and θ represents the incident angle of source. Ground (Ground plane) and Spacer heights are 150 nm and 60 nm respectively.

In the proposed tungsten based absorber for visible regime, the structure contains a ground plane made up of a metal underneath a dielectric layer which in turn is below a resonating structure made up of a same metal as ground plane (metal-dielectric-metal) as shown in Fig. 1. The dielectric layer is made up of silicon dioxide (SiO_2_). Whereas, both metal layers are made up of tungsten. The SiO_2_ is chosen because it has fairly low relative permittivity at optical range and, this more or less remains constant. The SiO_2_ also provides fairly high melting point which is a desired property for the dielectric layer as well.

The higher temperature withstanding capability (due to tungsten) of the absorber helps to better its efficiency in STPVs where, a concentrated light beam is shone on the hot layer to make temperature difference by absorbing large quantity of energy. Usually, a nanostructure on top gets disfigured with the application of concentrated beam of higher energy when using conventional materials such as Gold (Au) or Silver (Ag)^27^. Tungsten (W), attributing to its melting point, will withstand higher energy concentrated beams. This advantage of increased stability in elevated temperatures can be used in terrestrial applications, especially near close orbit to the sun^2^.

A design of square ring structure is taken here as an initial point. After the optimization of square ring structure, it is observed that the structure gives negligible reflectance for a cross-shaped configuration of nanostructure layer. The cross structure is then analyzed further in detail.

## Results and Discussions

### Four rods (square structure) simulations

Simulations of the structures are performed in a step by step fashion in Lumerical FDTD solutions. Material properties in the software are explored in detail and curve fitting is performed for tungsten (Palik) and SiO_2_ (Palik) for experimental values in^30^. Numbers of maximum coefficients are varied against permittivity which resulted in 6 coefficients for SiO_2_ and 15 coefficients for tungsten, as they best fitted the curve for optical domain. A structure of metal-dielectric (spacer)-metal (ground plane-GP) of a square ring is formed initially as a starting point as shown in Fig. 1.

When the rod is parallel to x-axis, its y-span is considered as its width, and when it is parallel to y-axis its x-span is considered as its width. If the rods are to make a square structure they must be placed at 100 nm, when center of the structure is at origin (0, 0). That is to say that the right rod will be at x = 100 nm from origin, left rod at x = −100 nm, top rod at y = 100 nm and bottom rod at y = −100 nm.

The mesh refinement is set to conformal variant 1 as it includes metal boundaries. The results shown in this paper are described with three types of mesh step settings [MSSs (c.f. supplementary Table S1) to decrease overall time for simulation.

**Figure 2:**
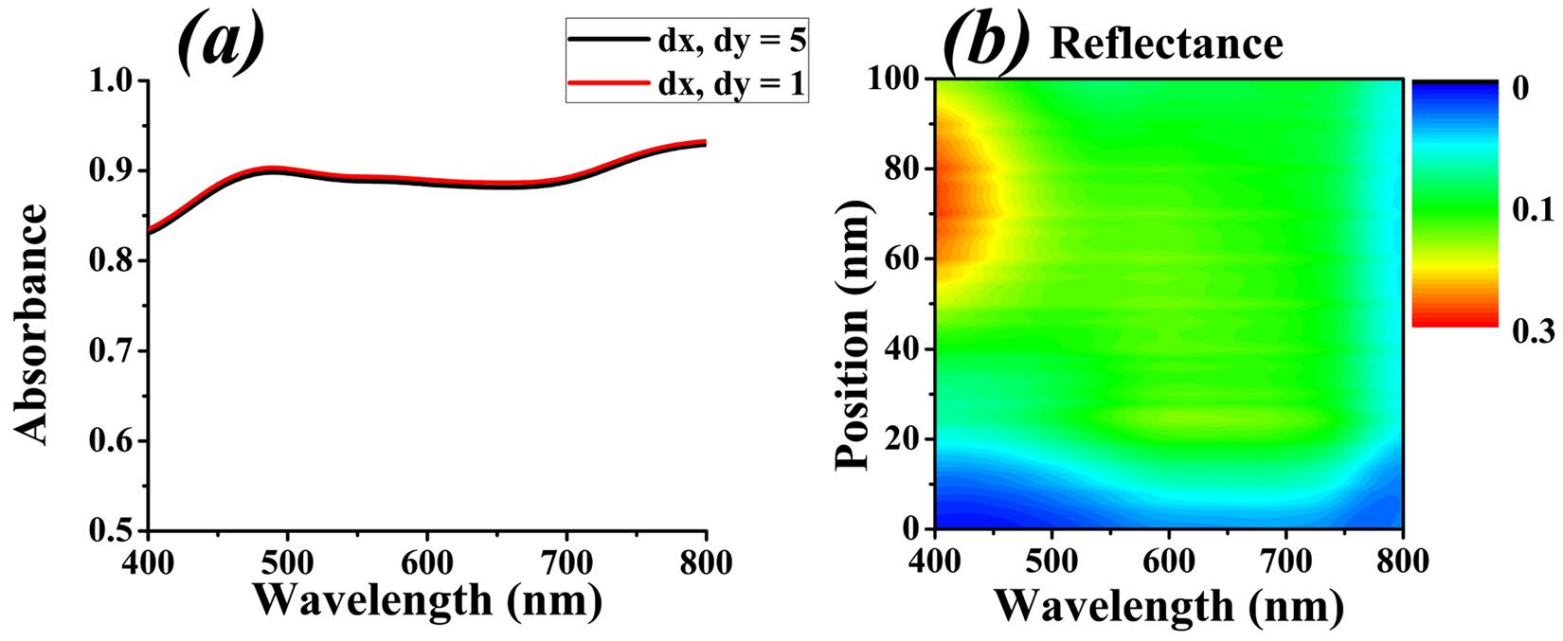
(**a**) Different mesh settings for rod structure. (**b**) Reflectance when changing position.

Simulation of this square ring structure resulted in two different absorbance curves for two maximum mesh step settings (MSS 1 and MSS 2) as shown in Fig. 2(a).

The results lead to two important conclusions (i) the square-shaped structure is not impedance matched with the free space and (ii) the mesh step settings play a little role in absorbance curve. The results for absorbance are calculated from equation 1, as simulations give reflection and transmission.

1 $$A=1-|T|-|{\rm{\Gamma }}|$$

where, A is absorbance, T represents transmission, and Γ shows reflection. The equation 1 can also be manipulated in terms of s-parameters, given by equation 2.

2 $$A=1-{|{S}_{12}|}^{2}-{|{S}_{11}|}^{2}$$

### Optimization of Rods for Tungsten

**Figure 3:**
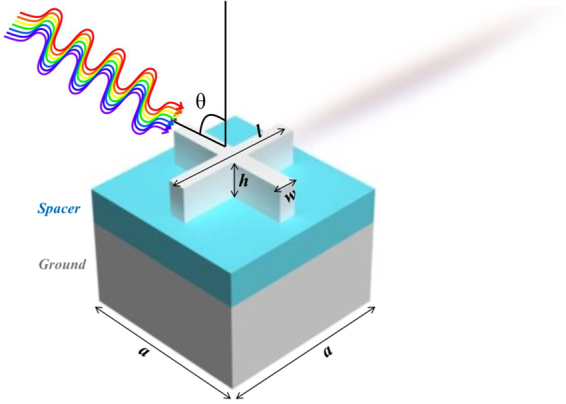
Cross-shaped design for tungsten where *w* is width, *h* is height and *l* is length and θ represents the incident angle of source.

As the structure of rods is not impedance matched for tungsten, variations are made, keeping in mind that the structure still remains polarization insensitive. Optimization reveals that if the rods are moved towards opposite direction (i.e., top rod is moved towards bottom and vice versa, and similarly, right rod towards left rod and vice versa), the structure still remains polarization insensitive but its effective parameters such as impedance (z), refractive index (n), permittivity (ε_r_) and permeability (µ_r_) vary. We named these effective parameters as figures of merit (FOM). By changing the front nanostructure, the device’s impedance varies and reflection is observed, by altering the displacement of rods over the entire optical range as exhibited in Fig. 2(b). It also shows that the rods are moved only 100 nm towards the origin (0, 0) i.e., right rod is moved 100 nm to the left and vice versa, because by symmetry, further displacement would result in the same plot (except inverted along the horizontal direction). Furthermore, the results elaborate that the structure gives minimum reflection with cross-shaped configuration (like in Fig. 3). Therefore, optimum design for the tungsten-based absorber is proposed as cross-shaped as depicted in Fig. 3.

**Figure 4:**
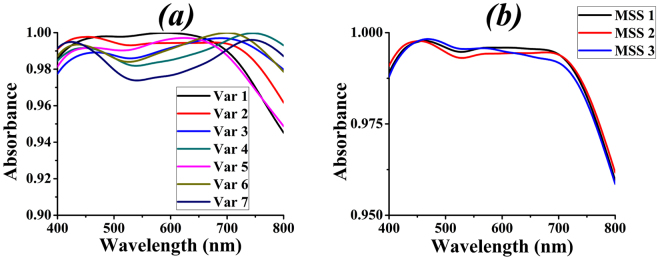
(**a**) Different variations of cross design using MSS 2. (**b**) MSS configurations for cross.

The cross-shaped structure can have different configurations by varying its structural parameters such as width (*w*), height (*h*) and length (*l*) which alters overall impedance of the device. Variations in cross structure are made by changing the aforementioned parameters and results of absorbance are plotted in Fig. 4(a). Using these results, average absorbance due to parametric variations (Var 1-Var 7) are calculated by summing absorbance of all points divided by total number of points shown in Supplementary Table S2. The MSS 2 is used for attaining results in Fig. 4(a) and only the best results are displayed i.e., with 98% absorbance as a minimum threshold. Results show that Var 2 (i.e., cross-shaped structure having w = 30 nm, h = 60 nm and l = 225 nm) yields the best absorbance, hence these values are taken as design values.

Different mesh step settings are applied to the design Variation 2 (Var 2) and absorbance curves are attained to see if MSS 2 is giving comparable results as shown in Fig. 4(b). Var 2 is plotted as it is taken as design variation.

The results in Fig. 4(b) show an average absorbance of 99.3455%, 99.3141% and 99.2943% for MSS 1, 2 and 3 respectively. This shows that the MSS 1, 2 and 3 can be used interchangeably as they do not produce significant error but using greater step settings reduces the simulation time. Therefore, for the rest of the results shown in the paper MSS 2 is used.

To understand the phenomenon of high absorbance in this design, electric-fields are monitored from x-y plane (just above the cross-shaped structure), x-z plane (cut in the center of the structure) and y-z plane for four wavelengths (λ = 400 nm, 500 nm, 600 nm and 700 nm) as shown in supplementary Fig. S2. These images exhibit a strong field-localization for higher wavelengths (such as λ = 700 nm and 800 nm) and it reduces as the wavelength decreases, as depicted in supplementary Fig. S2. This can be further elaborated by ${P}_{abs}=\frac{1}{2}\omega \varepsilon ^{\prime\prime} {|E|}^{2}$.

The results of field-localization indicate a pattern of absorbance of the proposed absorber keeping the imaginary part of relative permittivity of tungsten in mind (shown in supplementary Fig. S1). The imaginary part of relative permittivity of tungsten increases from 16 to 22 and for 400–700 nm (approximately) and then decreases to about 19 at 800 nm as shown in supplementary Fig. S1. In effect, the contribution of the front-layer of the cross-shaped should become significant at 700 nm, but this contribution should give a decreasing trend after 700 nm (suggested by the trend of permittivity) which is confirmed by find P_abs_ for this absorber provided by supplementary Fig. S3. In order to investigate the previously developed hypothesis by simulations, the contributions of front-layer (cross-shaped nanostructure and SiO_2_ layer) and back-layer (ground plane of Tungsten) to the absorbance are established by simulation. Close observance suggests that if absorbance from one layer and total absorbance is known, then the absorbance from unknown layer is calculated by subtracting the two. In our case, the total absorbance and back-layer’s absorbance are calculated via simulation and front-layer’s contribution is taken by subtracting. Division of the absorber into two layers is made on the basis of two phenomena of absorption working in parallel i.e. intrinsic dielectric loss in back-layer and dielectric resonance due to front-layer.

**Figure 5:**
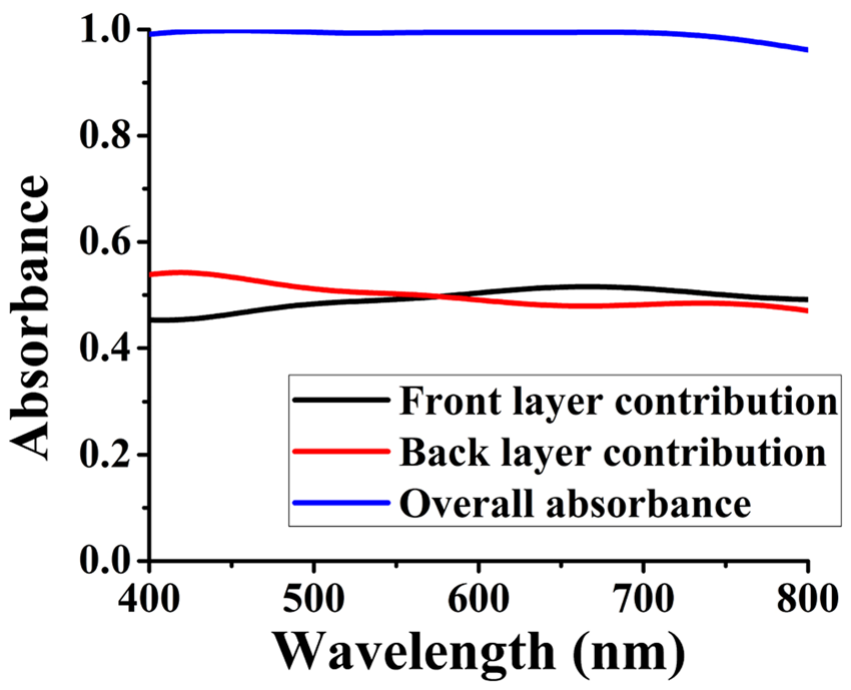
Front and back-layer’s contribution to absorbance.

The results in Fig. 5 show that the magnitude of contribution from both layers (front and back) is quite high and suggests that back-layer is contributing more towards the overall absorbance till λ = 575 nm but after that, front-layer’s contribution is comparably higher than the absorbance achieved by the back-layer. This suggests that the intrinsic loss inside due to back-layer is higher till λ = 575 nm but after that resonance becomes stronger and contributes more towards absorption also observed in supplementary Fig. S2. This elaboration helps us to understand the underlying phenomenon which is directly responsible for this ultra-high absorbance.

The resonance in the front-layer is further explored by taking one rod (rod of tungsten with length of 225 nm in x-direction and a rod of same length in y-direction) one at a time and simulating the structure with different polarizations i.e. x-polarization and y-polarization. The results presented in supplementary Figs S4–S7, show that the resonating bars give almost the same ultra-high absorbance for their respective polarization i.e. x-spanned bar for x-polarization and y-spanned bar for y-polarization but when simulated for the other polarization, the results diminish significantly. In order to keep the structure polarization insensitive, cross-shaped structure is chosen, which gives the same results for both polarizations.

**Figure 6:**
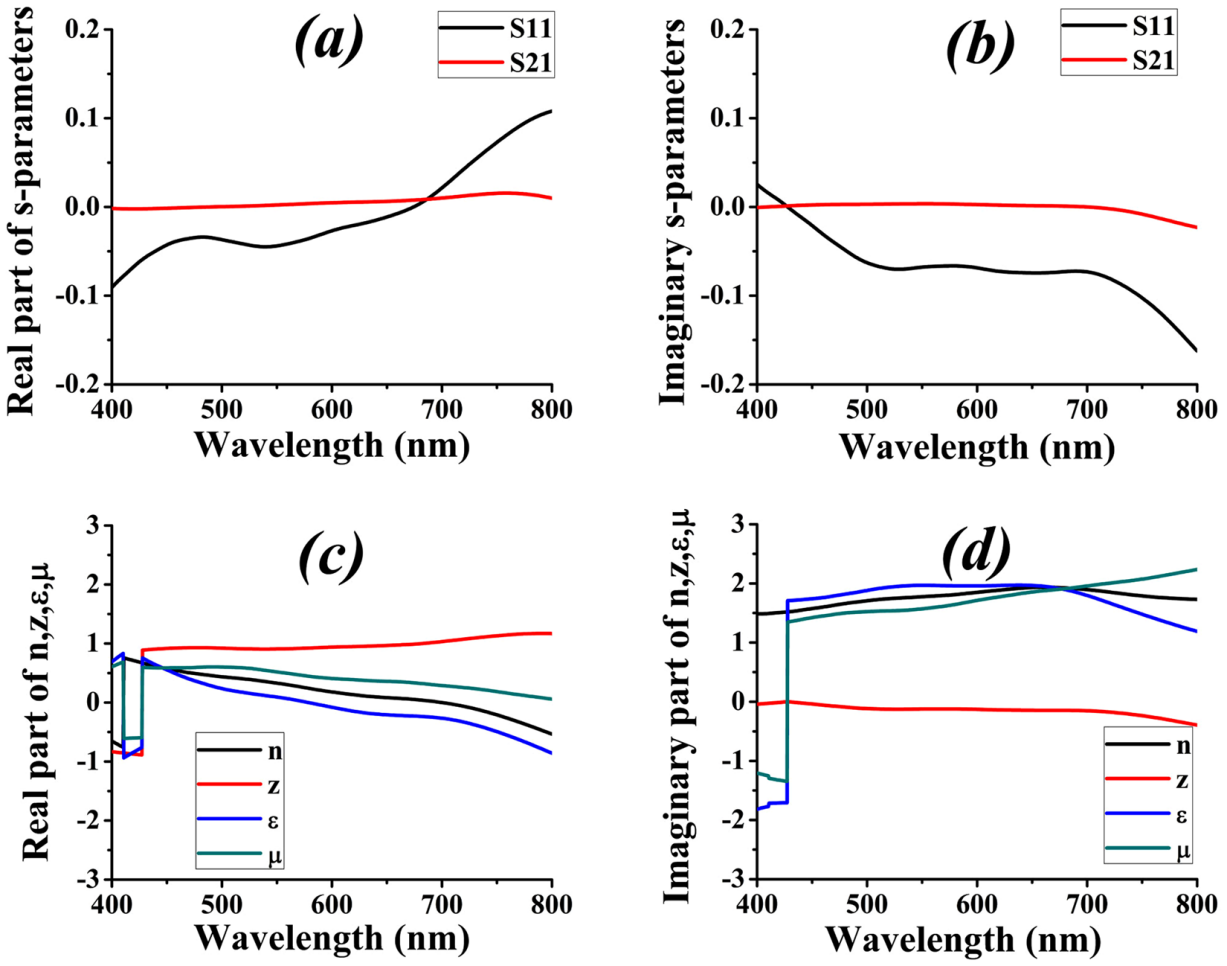
(**a**) Real part of s-parameters. (**b**) Imaginary part of s-parameters. (**c**) Real part of figures of merit. (**d**) Imaginary part of figures of merit.

This ultrahigh absorbance is achieved because the cross-shaped tungsten absorber is impedance matched with the free space. A theoretical proof of this observation can be attained if s-parameters of the structure are known. The s-parameters can also help to find the overall reflection and transmission and thus, absorbance by utilizing equation (2). In this regard, we have used analysis group of Lumerical FDTD solutions software to numerically extract the s-parameters of our device, the results of which are plotted in Fig. 6(a) and (b).

Once s-parameters are known, other related parameters such as impedance, refractive index, relative permittivity and relative permeability of the device can be calculated by using the equations^31–34^ mentioned below.

3 $$z=\pm \sqrt{\frac{{(1+{S}_{11})}^{2}-{S}_{21}^{2}}{{(1-{S}_{11})}^{2}-{S}_{21}^{2}}}$$

where,

z represents the impedance.

The effective refractive index of the overall structure (combination of front and back-layer) can be calculated as^31–34^.

4 $$n=\frac{-i\,\mathrm{ln}({e}^{in{k}_{o}d})}{{k}_{o}d}$$

where,

i = imaginary number and

d = the thickness of the absorber (its height).

In aforementioned equation, ${e}^{{in}{k}_{0}d}$ is defined as.

5 $${e}^{in{k}_{o}d}=X\pm i\sqrt{1-{X}^{2}}$$

where,

$$X=\frac{1}{2{S}_{21}(1-{S}_{11}^{2}+{S}_{21}^{2})}$$, and

${k}_{o}=\frac{2\pi }{\lambda }$ is the wave number of the free space.

To employ above mathematical expressions, the values of impedance and refractive index are chosen such that real-value of impedance and imaginary-value of the refractive index should be greater than 0^31–34^. After obtaining n and z, the effective permittivity (ε_r_) and the effective permeability (μ_r_) can be found by the following formulas^31–34^.

6 $${\varepsilon }_{r}=\frac{n}{z}$$

7 $${\mu }_{r}=nz$$

Figure 6(c) and (d) represents the real and imaginary plots of the figures of merits [FOMs; z, n, ε_r_ and μ_r_] that are obtained from the analysis group of Lumerical FDTD solutions [which uses equations (3) to (7) to plot them]. The software applies same condition to get “n” but selects negative value of complex component of “z”, which is slightly different procedure than the one mentioned earlier. For the sake of reproducibility only software results are shown here but corresponding conditional results, where real value of “z” greater than 0 is taken, are attached in supplementary Figs S7 and S8. These results clearly depict that the device is impedance matched with the free space as z almost remains unity for the optical regime. This factor alone establishes the ground for a perfect light absorber.

**Figure 7:**
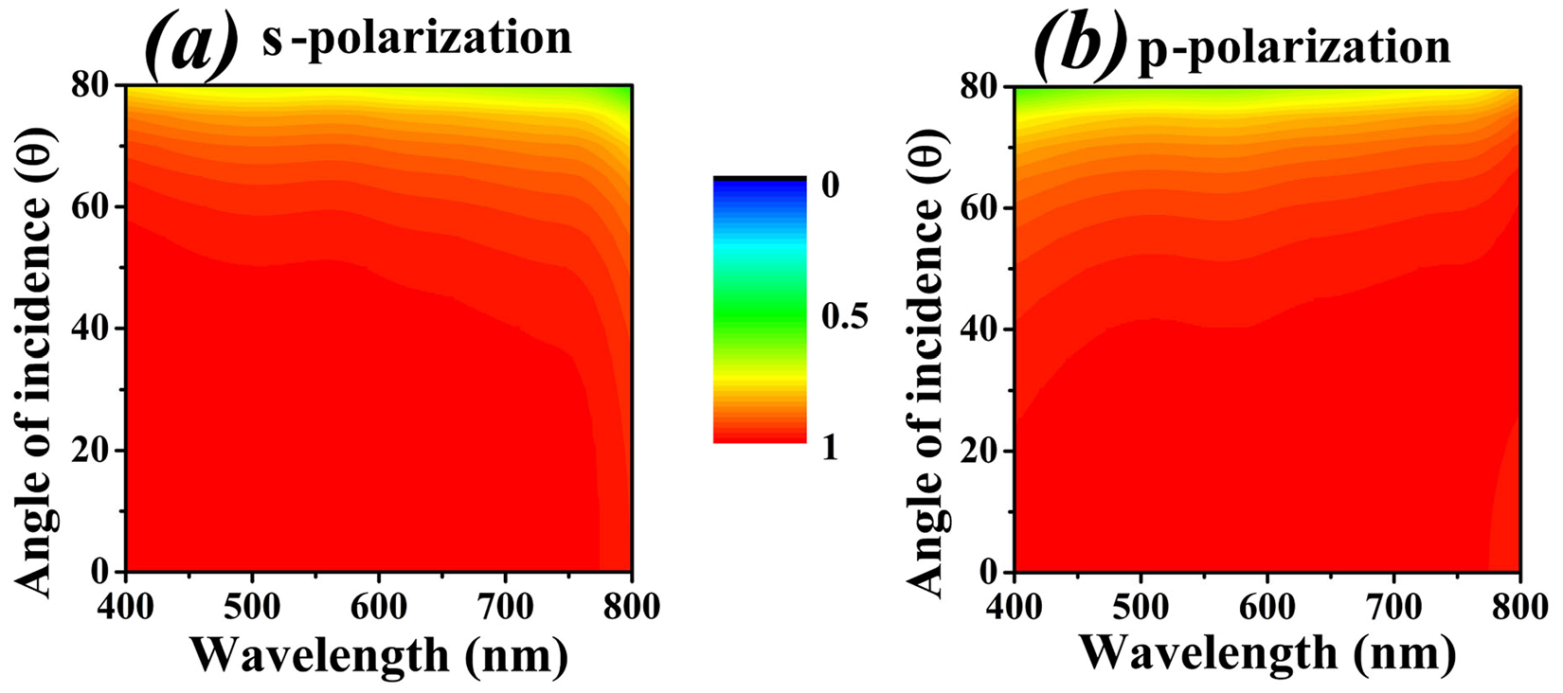
(**a**) Angle of incidence versus wavelength for s-polarized source. (**b**) Angle of incidence versus wavelength for p-polarized source.

Apart from being impedance matched, the performance of a good absorber should not be deteriorated due to variations in the incident-angle of the source. Therefore, the cross-shaped absorber is further simulated for s-polarized and p-polarized light to find out the variations in absorbance. The results of incident-angle θ (theta) with respect to wavelengths in visible regime are shown in Fig. 7(a) and (b), s-polarized and p-polarized respectively. The results in Fig. 7(a) and (b) show that the structure is highly optimized for sources, s and p polarized, with a greater angle of incidence. The structure gives almost a unity absorbance for angles as high as 70° (θ < 70°). For the angles greater than 70° (θ > 70°), the design loses its perfect absorbance in both cases of s and p polarized source.

**Figure 8:**
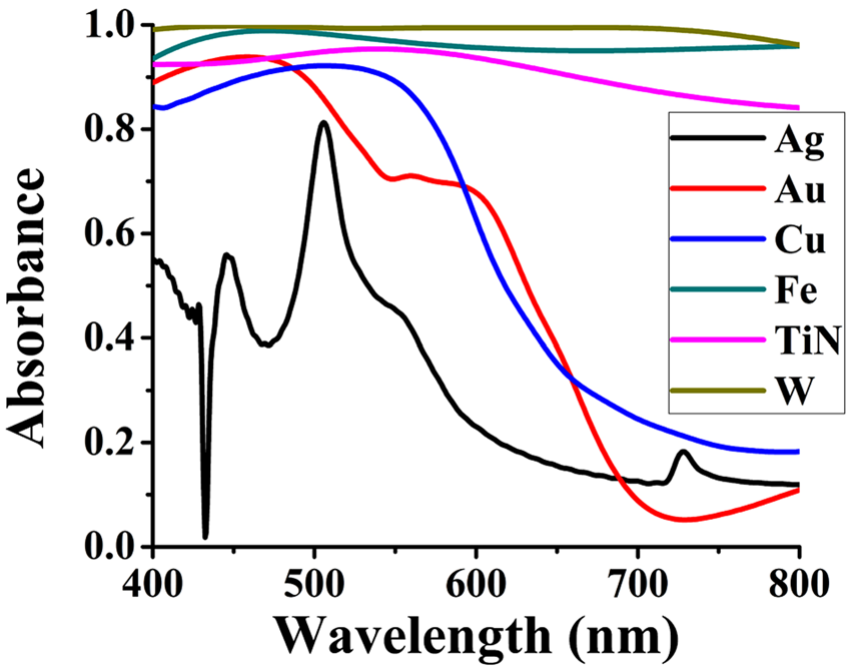
Absorbance achieved by various metals and TiN.

This cross-shaped structure is also compared for other metals (and refractory ceramic titanium nitride “TiN”) by replacing tungsten from nanostructure and ground plane without changing any other dimensions. The results are summarized in supplementary Table S3 and the response of these absorbers for visible regime is plotted in Fig. 8. The plot indicates that tungsten based configuration is best optimized for a cross-shaped absorber, though absorbance of iron “Fe” is close. Noble metals such as silver “Ag” and gold “Au” are not giving as much absorbance as that of tungsten.

The major principle of absorbance in other metals and TiN is impedance matching and surface plasmonic resonance^35,36^. This however, is not true for the case of tungsten-based structures as surface plasmons do not exist for tungsten in the visible spectrum^30,35^. Hence, this high absorbance of the proposed (tungsten-based) design is solely achieved due to its impedance matching with free space which can be confirmed by front-layer’s resonance and back-layer’s intrinsic loss.

### Integration of absorber in photovoltaics

An effective STPV system and solar thermoelectric generator (STEG) consists solar absorber amongst other components^2^. The provided tungsten based absorber is for solar absorber part of STPV and STEG. An efficient absorber can also be made for photovoltaic cell which employs the phenomenon of electron-hole pair generation, by changing the substrate (SiO_2_) in W-SiO_2_-W structure presented in this paper into a semiconductor material such as silicon (Si). A highly optimized structure for integration into photovoltaic cells is presented in supplementary table S4 in section F. Electron-hole pair will be generated in silicon layer and collected at top nanostructure layer.

## Conclusion

Highly efficient and flat optical nanoabsorber based upon tungsten (having ultra-high melting point of 3422 °C) is investigated. The proposed ultrathin absorber outperforms previously reported metal-based absorbers in terms of efficiency, operational spectrum and melting point, resulting in higher stability of cross nanostructure. The symmetrical geometry of the cross makes it polarization insensitive which is a promising attribute of the absorber. The significant absorbance is achieved by this design due to its impedance matching with the free space which can also be explained by intrinsic loss in back-layer and resonance in front-layer. Further investigations reveal that the presented absorber achieves high absorbance for the sources which are incident at an elevated angle theta (θ < 70°).

### Availability of materials and data

The authors impose no restriction in reproduction of the design and all data is provided inside the manuscript.

## Electronic supplementary material

Supplementary Information
