# Supplementary material for: Tungsten-based Ultrathin Absorber for Visible Regime
Source: Sci Rep. 2018 Feb 5;8:2443. doi: 10.1038/s41598-018-20748-9 (PMC5799306; doi:10.1038/s41598-018-20748-9)
Supplement: Supplementary file 1 — Supplementary Information [file 41598_2018_20748_MOESM1_ESM.doc]

# Tungsten Based Ultrathin Absorber for Visible Regime

(Supplementary Information)

Ahsan Sarwar Rana^1^**^†^**, Muhammad Qasim Mehmood^1,2^**^†^**⋆, Heongyeong Jeong^2^, Inki Kim^2^, Junsuk Rho^2,3^⋆

^1^Department of Electrical Engineering, Information Technology University of the Punjab, Lahore 54000, Pakistan. Tel: +923360502237; ⋆Email: [qasim.mehmood@itu.edu.pk](mailto:qasim.mehmood@itu.edu.pk),

^2^Department of Mechanical Engineering, Pohang University of Science and Technology (POSTECH), Pohang 37673, Republic of Korea. ⋆Email: jsrho@postech.ac.kr

^3^Department of Chemical Engineering, Pohang University of Science and Technology (POSTECH), Pohang 37673, Republic of Korea.

**^†^** These Authors contributed equally to this work.

**Key Words**: Tungsten, Sub-wavelength, Absorbance, Ultrathin, Metasurface.

RECEIVED DATE

1. **Mesh Step Settings (MSS)**

Table S1: Mesh step settings (MSSs)

| Mesh Step Setting | dx | dy | dz |
| --- | --- | --- | --- |
| MSS 1 | 1 | 1 | 5 |
| MSS 2 | 5 | 5 | 5 |
| MSS 3 | 10 | 10 | 10 |

1. **Curve Fitting and extraction of P_abs_ for tungsten**

The curve fitting of experimental data ^1^ is performed in Lumerical FDTD solutions for tungsten and SiO_2_. FDTD model is attained for tungsten and SiO_2_ by selecting their coefficients as 15 and 6, respectively, and fit tolerance is set to 0. Figure S1 shows the curve fitting of tungsten along with its FDTD model.


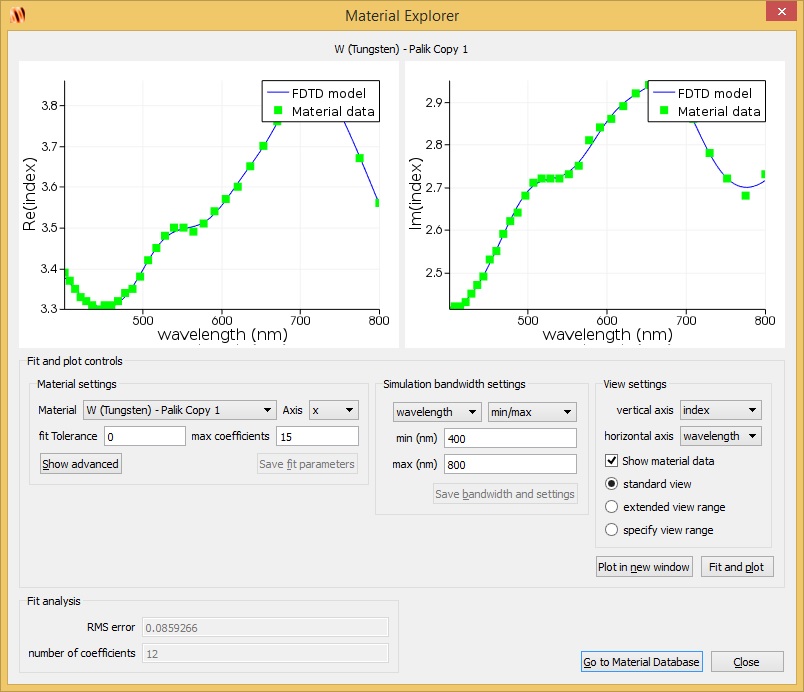


Figure S1: Curve fitting for tungsten

The electric field profiles (|E|^2^) of the absorber as seen from x-y plane, x-z plane and y-z plane are shown by the help of Fig. S2. It can be establishes that the x-bar contributes more towards absorbance if the source is x-polarized and y-bar contributes more when source is y-polarized.


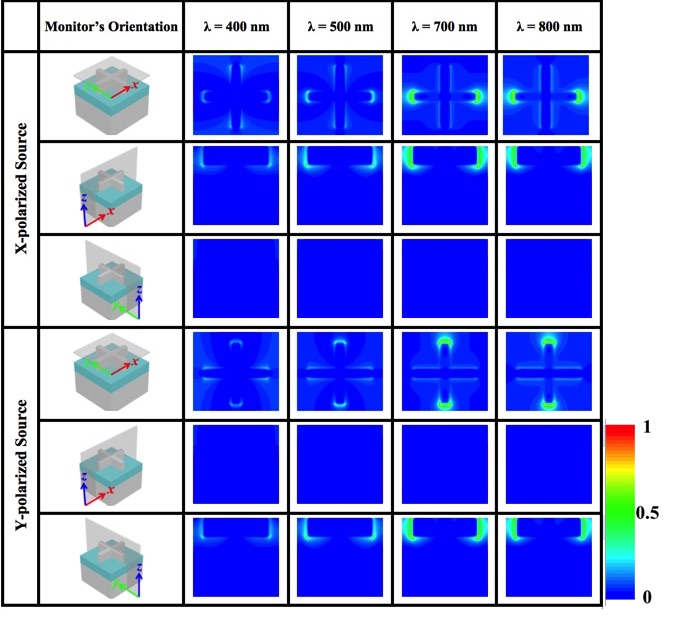


Figure S2: |E|^2^ profiles for different cut planes (x-y, x-z and y-z) observed for different polarizations (x-polarization and y-polarization) at different wavelengths (400 nm, 500 nm, 700 nm and 800 nm).

This FDTD model developed in Fig. S1 is used to extrapolate values of imaginary permittivity which is further used to analyze power absorber in the absorber using$P_{abs}=\frac{1}{2}\omega\varepsilon"\left|E\right|^{2}$. Absolute squared electric-field is taken by summation of electric-field profiles (as in Fig.S2 (a)) at various wavelengths. Figure S2 shows the P_abs_ versus wavelength for optical regime for this cross-shaped tungsten absorber.


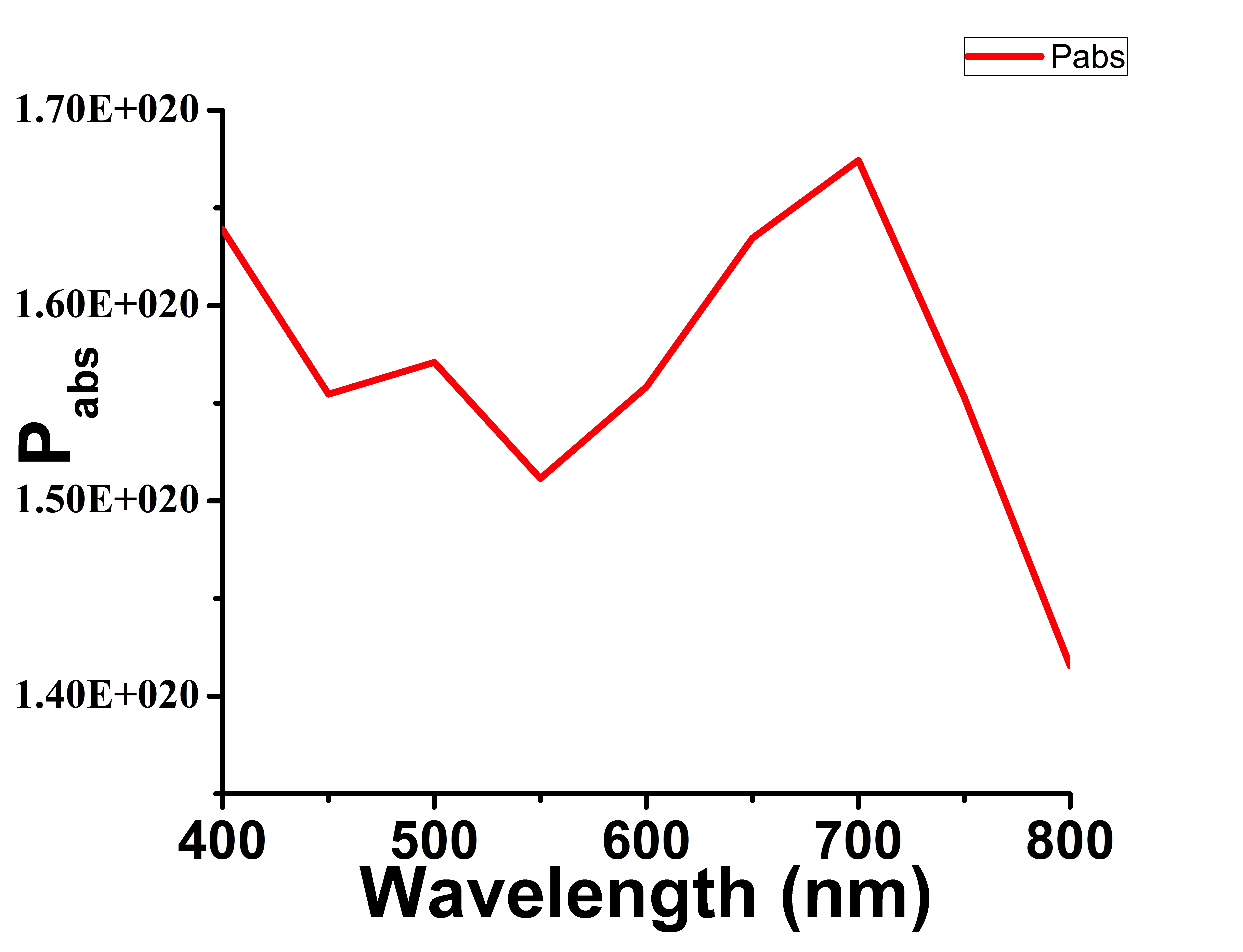


Figure S3: P_abs_ versus wavelength for optical regime

1. **Cross-shape variations**

The results show that variation 2 (Var 2) gives the best absorbance; therefore, these parameters are taken as a design.

Table S2: Different simulation results

| # | Structure dimensions | | | Absorbance (%) |
| --- | --- | --- | --- | --- |
|  | Width (nm) | Height (nm) | length (nm) |  |
| Var 1 | 30 | 60 | 200 | 99.2615 |
| Var 2 | 30 | 60 | 225 | 99.3141 |
| Var 3 | 30 | 80 | 200 | 98.8946 |
| Var 4 | 30 | 80 | 225 | 98.9047 |
| Var 5 | 40 | 60 | 175 | 98.8504 |
| Var 6 | 40 | 60 | 200 | 99.0928 |
| Var 7 | 40 | 60 | 225 | 98.5422 |

1. **Absorbance in top layer**

Absorbance in structure due to dielectric resonance in top layer, corresponding to each bar of cross with varying polarizations is shown below.


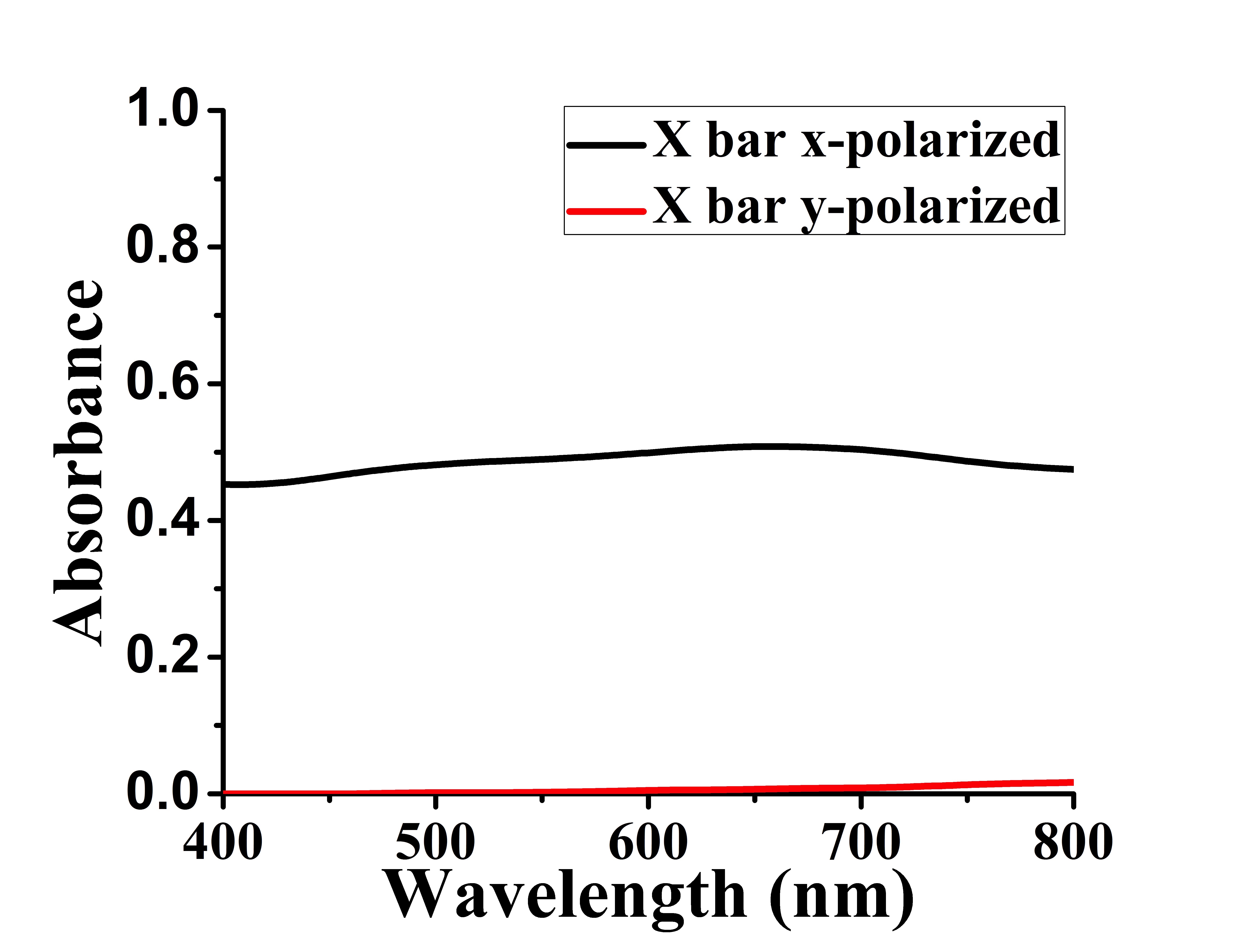


Figure S4: Absorbance in x-bar due to x and y polarizations.


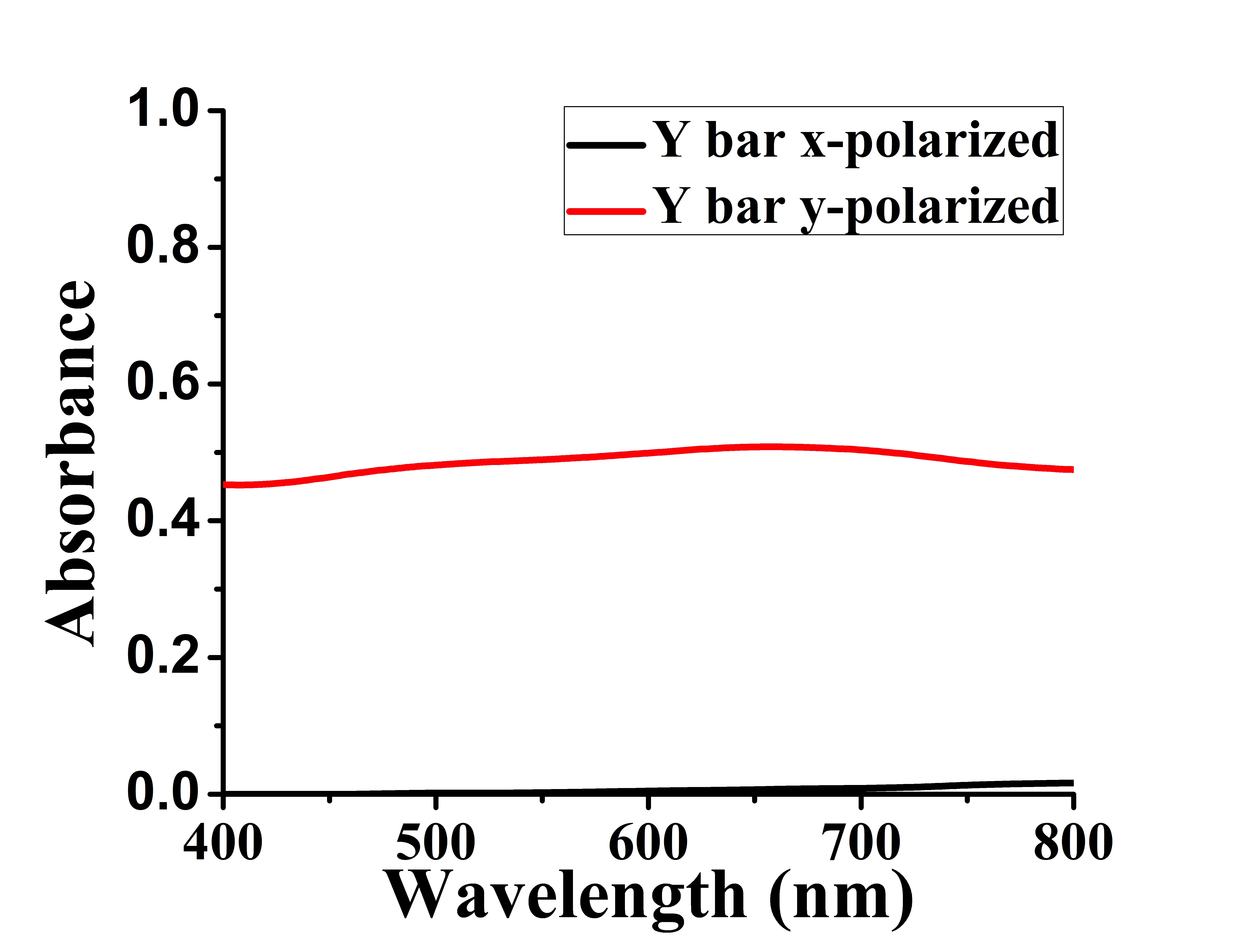


Figure S5: Absorbance in y-bar due to x and y polarizations.


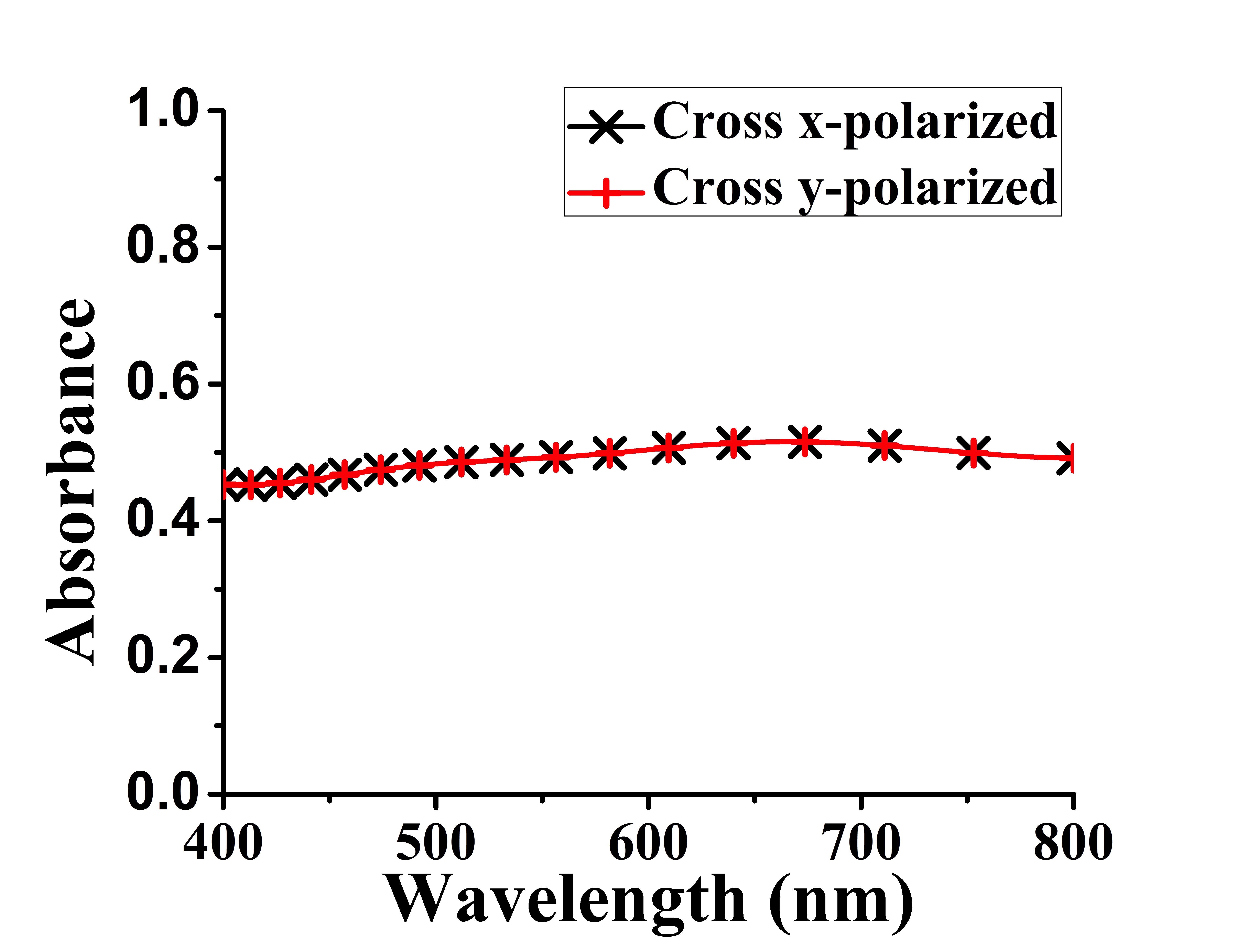


Figure S6: Absorbance in cross due to x and y polarizations.

1. **Figures of Merit**

Figures S4 and S5 show the results obtained by taking real value of “z” and imaginary value of “n” greater than 0.


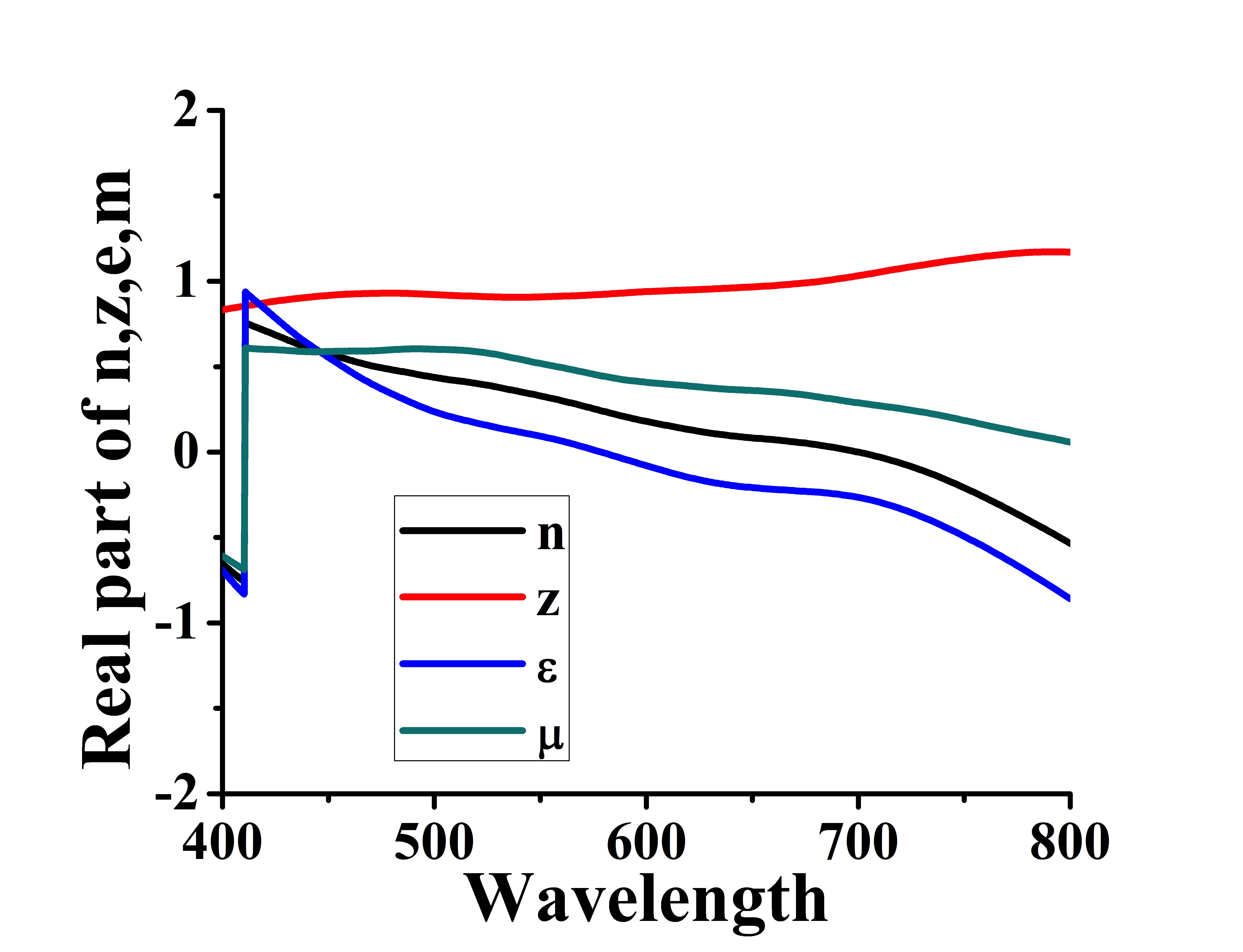


Figure S7: Real part of figures of merit.


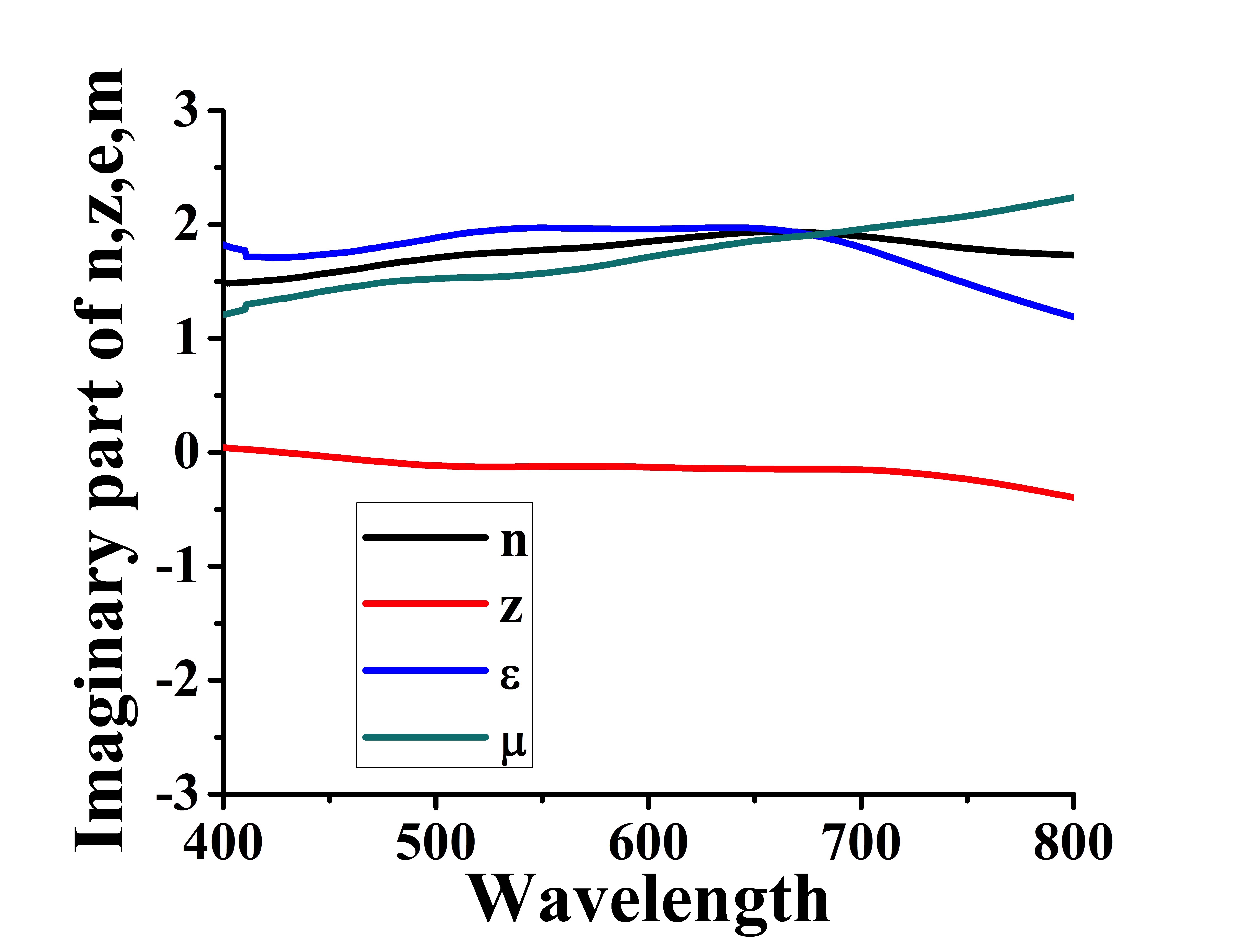


Figure S8: Imaginary part of figures of merit.

1. **Absorbance achieved by different metal configurations on the same geometry**

Table S3: Absorbance by different metals and refractory ceramic

| Metal | Curve fitting (max. Coefficients) | Absorbance (%) |
| --- | --- | --- |
| Silver (Ag) | 6 | 36.3412 |
| Gold (Au) | 9 | 65.255 |
| Copper (Cu) | 8 | 68.4969 |
| Iron (Fe) | 6 | 96.6325 |
| Titanium Nitride (TiN) | 6 | 92.1098 |
| Tungsten (W) | 15 | 99.3141 |

1. **Optimized dimensions for photovoltaic system**

Optimized structural parameters for W-Si-W and corresponding absorbance are provided in the table S4. Figures S6 exhibits the definitions of various structural parameters such as height of the spacer ***h1***, height of the nanostructure ***h2***, length of the cross nanostructure ***l*** and width of the cross ***w***. The period of the absorber (***a***) is kept the same at 300 nm.


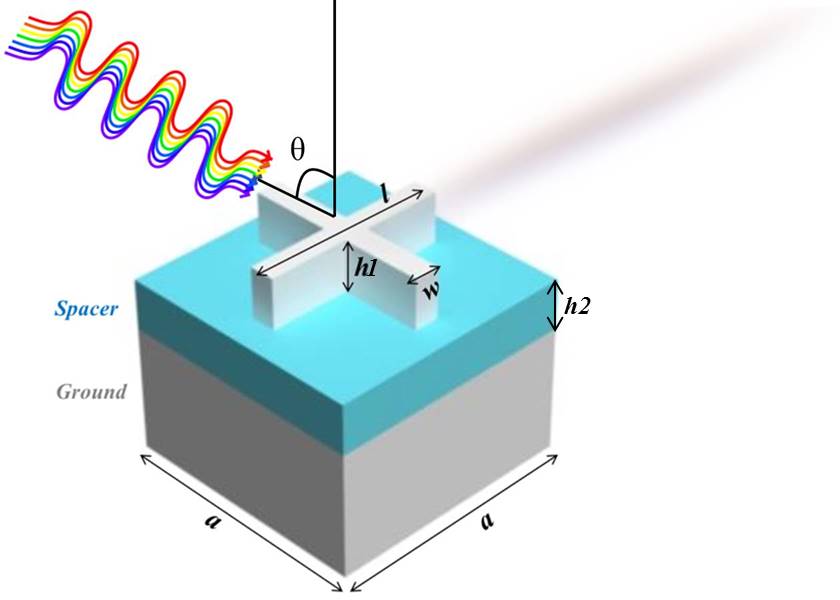


Figure S9: Tunsgten based design for Silicon.

Table S4: Optimized dimensions of W-Si-W structure.

| Results | *h*1 nm | *h*2 nm | *l* nm | *w* nm | Absorbance (%) |
| --- | --- | --- | --- | --- | --- |
| Result 1 | 262 | 169 | 220 | 44 | 97.355 |

References

1 Palik, E. D. *Handbook of optical constants of solids*. Vol. 3 (Academic press, 1998).
